# Recommendations for the Role of Publishers in Access to Data

**DOI:** 10.1371/journal.pbio.1001975

**Published:** 2014-10-28

**Authors:** Jennifer Lin, Carly Strasser

**Affiliations:** 1 PLOS, San Francisco, California, United States of America; 2 California Digital Library, University of California, Oakland, California, United States of America

## Abstract

As appeals for public access of research data continue to proliferate, many scholarly publishers—alongside funders, institutions, and libraries—are expanding their role to address this need. Here we outline eight recommendations and a set of suggested action items for publishers to promote and contribute to increasing access to data. This call to action emerged from a summit that brought together data stewardship leaders across stakeholder groups. The recommendations were subsequently refined by the community as a result of public input gathered online and in meetings.

## Abstract 2

This community perspective piece calls on publishers to promote and contribute to increasing access to data in their role with eight simple recommendations and example action items.

## Background

Institutions that support research have a vested interest in preserving and promoting the work of their researchers. This work includes scholarly publications, software, datasets, reports, and other outputs. A 2013 memorandum from the White House Office of Science and Technology Policy (http://www.whitehouse.gov/administration/eop/ostp) requires that government funding agencies ensure all research output that results from work they support be publicly available. In the United Kingdom, Research Council policies (http://roarmap.eprints.org/671/1/RCUK%20_Policy_on_Access_to_Research_Outputs.pdf) require that data be made available and preserved for 10 years and that research publications contain a statement on how the underlying materials—such as data, samples, or models—can be accessed. More widely, the European Union Horizon 2020 program (http://ec.europa.eu/programmes/horizon2020/) includes an Open Research Data pilot (http://europa.eu/rapid/press-release_IP-13-1257_en.htm) that will require data sharing of grantees.

These new policies have significant repercussions: stakeholders such as institutions and funders will need to provide researchers with the means to preserve and provide access to their research outputs. At the same time, librarians, information technologists, preservation specialists, and others have a long history of providing infrastructure, education, and support for preserving and promoting researchers' outputs. These new policies only bolster the importance of their efforts as they relate to data [1],[2]. Publishers are also a critical stakeholder group as the current “gatekeepers” of formal scholarly research and, increasingly, of other research outputs beyond the research article.

Given this climate of new mandates, changing roles, and increasing challenges, we convened a meeting with a group of leaders in data stewardship to discuss, “What can publishers do to promote the work of libraries and institutions in advancing data access and availability?” The event coincided with the International Digital Curation Conference on February 26, 2014. A diverse group of data experts was present (Box 1), including repository heads, librarians, funders, infrastructure builders, program directors, developers, and researchers. The group developed a range of priorities and recommendations for publishers. To allow attendees to establish a common voice and brainstorm freely, publishers were intentionally not included in the discussion (individuals affiliated with PLOS were present only in the capacity of hosts and facilitators).

**Box 1. Contributors of the Role of Publishers Meeting on February 26, 2014**

**Participants**

- Stephen Abrams, Associate Director of University of California (UC) Curation Center, California Digital Library
- Rachel Bruce, Director, Technology Innovation, JISC
- Eleni Castro, Research Coordinator, Institute for Quantitative Social Science (IQSS), Harvard University
- Patricia Cruse, Director of UC Curation Center, California Digital Library
- Ingrid Dillo, Head Policy Communication Development, Data Archiving and Networked Services (DANS)
- Alex Garnett, Data Curation and Digital Preservation Specialist, Simon Fraser University
- Jennifer Green, Director of Research Data Services, University of Michigan
- Simon Hodson, Executive Director, CODATA
- Eric Kansa, Technology Director, Open Context
- Belinda Norman, Research Data Manager, University of Sydney
- Mark Parsons, Secretary General, Research Data Alliance
- Jonathan Tedds, Senior Research Fellow, University of Leicester
- Todd Vision, Principal Investigator, Dryad; Associate Director for Informatics, National Evolutionary Synthesis Center

**Hosts**

- John Chodacki, Director of Product Development, PLOS
- Jennifer Lin, Senior Product Manager, PLOS
- Cameron Neylon, Advocacy Director, PLOS
- Carly Strasser, Data Curation Specialist, California Digital Library

The outcomes of this summit were then submitted to the community for comment. The public solicitation for input was detailed in two blog posts by PLOS (http://blogs.plos.org/tech/feedback-wanted-publishers-data-access/) and the California Digital Library (CDL) (http://datapub.cdlib.org/2014/03/24/feedback-wanted-publishers-and-data-access/) and promoted across social media outlets. An additional feedback session was held at the Third Plenary of the Research Data Alliance (RDA) on March 28, 2014, the largest gathering of the international data community. The report below presents the public endorsements, which have been amended, validated, and refined over the course of 2.5 months by the community at large. While this effort was intentionally designed to speak to publishers, we encourage companion efforts to establish community-based endorsements aimed at other critical stakeholders in the research ecosystem.

## Call to Action

As a community, we envision a future information ecosystem in which research data is considered an integral part of scholarly communications. We propose a new metaphor for this vision: a social contract. This contract is an agreement amongst all stakeholders based on shared governing principles: data should be preserved, discoverable, measured, and integrated into evaluation processes, and data sharing is a fundamental practice. Adherence to this social contract will entail dramatic changes to existing workflows, technologies, and social norms for all the members of the research ecosystem.

While data stewardship requires expertise and knowledge that will be spread across other stakeholder groups (data centers, researchers, librarians, etc.), this document addresses the potential role of publishers in promoting the collective vision. Publishers play a critical role in this collective space, be they commercial, nonprofit, society, open access, institutional, etc. Because of the importance of formal publications in the academic incentive structure, they occupy a leverage point in the research process. We see an opportunity for them to become a strong force in effecting social and technical change. They can serve as the implementation and/or enforcement arm at the point of publication for the governing principles mentioned above. They have the potential to serve as honest brokers, listening to concerns from institutions and libraries about issues concerning data curation and publication and engaging with the stakeholders to help establish and enforce agreed-upon standards that suit the community as a whole and ensure access to data underlying the works they publish. Publishers can strive to be honest and transparent about their services and the costs of those services, especially if data archival costs are incurred. Above all, they can collaborate and coordinate their efforts with repositories and funders to cement the principles of data sharing and reuse as mutual stewards of this new ecosystem.

## Recommendations

Collectively, we recommend a comprehensive approach that encompasses the entire research process. We present eight action items for publishers to promote the work of libraries and institutions in advancing data preservation and access (Box 2). These are illustrated with concrete examples of projects that would support the high-level recommendations.

**Box 2. Recommendations for Publishers to Increase Access to Data**

1. Establish and enforce a mandatory data availability policy.
2. Contribute to establishing community standards for data management and sharing.
3. Contribute to establishing community standards for data preservation in trusted repositories.
4. Provide formal channels to share data.
5. Work with repositories to streamline data submission.
6. Require appropriate citation to all data associated with a publication—both produced and used.
7. Develop and report indicators that will support data as a first-class scholarly output.
8. Incentivize data sharing by promoting the value of data sharing.

### 1. Establish and enforce a mandatory data availability policy

The incentive structure for scholars is currently based on publishing journal articles: frequent publication, especially in high-impact journals, is perceived as a reliable indicator of a successful academic researcher for most disciplines [3]. Regardless of whether this incentive structure is ideal, it means that publishers are important gatekeepers in communicating science. In this role, publishers have a unique opportunity to effect change by requiring that data supporting the results of a publication be openly and freely available, by default. We recognize that there are some cases in which this is not possible due to privacy, sensitivity, or ownership issues, but these are exceptional cases and should be treated as such.

Not only should there be a policy in place, but it should be enforced. Many publishers “request” or “strongly recommend” that researchers make data openly available, but these policies are perceived as optional and rarely result in data availability. To this end, we recommend that the policy be applied as a mandatory one. Vines et al. [4] found that mandated archiving policies increased the odds of finding associated data by almost 1000-fold. This suggests that by establishing and enforcing a data policy, publishers can have a dramatic effect on data availability.

Examples of projects to implement include the following:

- Establish a searchable registry of journal data policies. Build off of previous work by the Joint Information Systems Committee (JISC)-funded pilot Journal Research Data Policy Bank (JoRD) project (http://jordproject.wordpress.com).
- Promote the use of standardized data availability statements within articles.
- Include data availability statements as part of the peer review and production checklists and enforce through rejection of manuscript if criteria have not been met.
- Establish an efficient and effective process for enforcing data policy noncompliance when brought to light after publication. Publicly specify the enforcement mechanisms for full disclosure to authors prior to publication.

### 2. Contribute to establishing community standards for data management and sharing

We recognize that sharing data is not necessarily a simple or straightforward endeavor. Many questions arise, such as how to archive large datasets, how to handle sensitive data, which stage of the data should be shared (e.g., raw or processed), which repositories are acceptable for housing the data, and how long the data should be available [5],[6]. Publishers can contribute to the community discussions that determine these community norms and subsequently set and enforce journal policies that contribute to the grander community vision.

Examples of projects to implement include the following:

- Develop and promote common guidelines through the Committee on Publication Ethics (COPE) and similar bodies, which also address compliance and enforcement.
- Work with other stakeholders (e.g., scholarly societies and funders) on the establishment of a registry of journal data policies regarding data sharing.

### 3. Contribute to establishing community standards for data preservation in trusted repositories

There are many repositories available for publishing datasets; these repositories vary in their data access and use policies, their procedures for preserving and maintaining datasets, and their data deposition requirements. Researchers will expect guidance on how to select an appropriate repository for their data, especially if the publisher mandates data availability. We recognize that publishers would not necessarily drive this discussion but rather prompt the community to choose and implement repository assessment guidelines to help researchers choose appropriate repositories for their data. Existing standards and guidelines for repositories include the Data Seal of Approval (http://datasealofapproval.org/en/), the repository selection process for Thompson-Reuters Data Citation Index (http://wokinfo.com//products_tools/multidisciplinary/dci/selection_essay/), and the Digital Curation Centre (DCC) Trusted Repositories Audit and Certification (TRAC) program (http://www.dcc.ac.uk/resources/repository-audit-and-assessment/trustworthy-repositories). These systems, combined with existing searchable databases for repositories (Re3Data [re3data.org] and DataBib [databib.org]), are potential starting points for a community standards discussion. Once standards are established by the community, publishers can then enforce them through journal policies.

Examples of projects to implement include the following:

- Provide input on repository certification standards and encourage use of certified repositories.
- Favor integration with repositories that comply with agreed-upon community standards.
- Provide information to authors about existing resources for selecting an appropriate data repository (e.g., databib.org, re3data.org, Biosharing, and DataONE).

### 4. Provide formal channels to share data

Because publishers operate as a connection point for the research narrative and its supporting outputs, they should expand their services to better deliver research data. Some publishers are already embracing the challenge. For example, F1000Research has partnered with the repository Figshare to provide inline viewing of associated datasets. Other publishers are launching journal titles specifically for datasets, such as Nature Scientific Data (http://www.nature.com/sdata/) and GigaScience (http://www.gigasciencejournal.com/). The concept of data publishing is still in flux [7] and under scrutiny [8]; however, this should not prevent publishers from seeking out new channels for researchers to share datasets. By providing new means to share data, publishers can help promote its importance and value as a scholarly output.

Examples of projects to implement include the following:

- Collaborate with cross organizational projects like Shared Access Research Ecosystem (SHARE).
- Allow the publication of papers that describe high-value datasets as a regular stream within existing disciplinary journals rather than segregating such papers into specialized data journals.
- Work with repositories on streamlining the payment of open-access charges for articles and data by authors and institutions.
- Develop guidelines (e.g., through COPE) for the operation of enclaves for sensitive data and promote their use for data that could otherwise not be made available for reuse.

### 5. Work with repositories to streamline data submission

We recommend that publishers work closely with existing repositories to allow researchers to seamlessly deposit their data alongside their article submission, with minimal effort on their part to ensure the article and data are appropriately linked. The point of interaction between repositories and publishers is a useful juncture for setting best practices for identifiers, metadata transfer, archival standards, licensing, and other aspects of data management that will support interoperability. It is in the interests of publishers (and data repositories) that such community norms evolve rapidly and efficiently, as this will enable the offering of well-integrated services. The Dryad Digital Repository (http://datadryad.org) has a long history of working closely with publishers to ensure integration of datasets with their associated articles; this successful partnership should serve as a model for future collaborations with repositories.

Examples of projects to implement include the following:

- Collaborate with repositories that are using application programming interfaces (APIs) with standard protocols (e.g., Simple Web-service Offering Repository Deposit [SWORD]) to create plugins (e.g., Open Journal System (OJS) Dataverse plugin) or add-ons to streamline the data publication process.
- Encourage manuscript processing system vendors to streamline the availability of data in repositories for the peer-review process.
- Work with repositories to develop requirements for allowing peer reviewers access to data.

### 6. Require appropriate citation to all data associated with a publication—both produced and used

The move towards formal citation of data has been growing. This is partly due to the current reward system, which counts citations as a metric for impact. If data are to be recognized as important outputs, then they should be cited in the same way that articles are cited. This includes reuse of existing data in the course of producing an article as well as data produced as a result of the work reported on in the article. Publishers can provide mechanisms and guidance for scholars to cite datasets. This guidance on citation may reference existing community recommendations, such as the Federation of Earth Science Information Partners (ESIP) guidelines for data citation (http://wiki.esipfed.org/index.php/Interagency_Data_Stewardship/Citations) or the Joint Declaration of Data Citation Principles (https://www.force11.org/datacitation). Any citation to a dataset should have appropriate resolvable identifiers to allow linking directly to datasets.

Examples of projects to implement include the following:

- Provide guidelines for data citation to authors based on Joint Declaration of Data Citation Principles (upgrade data citation to references section, use a generic citation format, etc.).
- Improve metadata standards for references to external datasets within Journal Article Tag Suite (JATS), ideally distinguishing data produced from data used.
- Encourage CrossRef to coordinate with DataCite on recording symmetric links between publication digital object identifiers (DOIs) and data DOIs in their metadata records.

### 7. Develop and report indicators that will support data as a first-class scholarly output

We recognize that the current reward system for academics recognizes publications and their attendant citations. In order for data to be treated as an important scholarly output, its impact must be measurable. A first step towards ensuring this recognition is providing access to reports on indicators of impact such as data downloads, use, reuse, citation, and other metrics. These types of indicators are known as “alternative” metrics (altmetrics), since they are only recently available due to the rise of increasingly digital scholarly communication. The altmetrics community is growing steadily, with increasing calls for changes in the way that researchers and their outputs are evaluated [9].

Examples of projects to implement include the following:

- Develop conventions for combining altmetrics (e.g., views, downloads, bookmarks, and citations) of articles with their associated datasets.
- Establish and test pilot metrics that capture the online activity surrounding data.

### 8. Incentivize data sharing by promoting the value of data sharing

There are many explanations for why researchers are resistant to data sharing; perhaps most prevalent is a fear of lost rights to or benefits from the data if they are made publicly available. In addition, making data available for use by others is potentially difficult and time consuming. Any culture shift will require that some individuals forge new paths in demonstrating the value and importance of data sharing. Publishers could reward these individuals by sponsoring competitions or rewarding prizes for most reusable data, best data paper, or more reproducible results. Competitions and prizes are emerging as an effective means of highlighting new ideas, celebrating technical achievements, and providing overall direction to the industry.

Examples of projects to implement include the following:

- Extend the incentive strategy of badges for open-data practices that has been piloted by Center for Open Science (http://centerforopenscience.org/journals/).
- Promote articles based on higher-than-average data reuse as well as higher-than-average article readership.
- Create a top 100 luminary list for data reuse to create an element of competition, profile, and also work out how to measure such things.

## Conclusions

Publishers have the opportunity to play an important role in promoting sharing of and access to research data. Alongside funders, institutions, and researchers, publishers can help to build a vibrant research ecosystem in which research data is publicly available for maximum reuse. The eight recommendations laid out here, with suggested action items, represent concrete ways in which publishers can help to usher in an era of widely available public research data.
